# Characterization of five newly derived canine osteosarcoma cell lines

**DOI:** 10.1186/s12917-019-2099-y

**Published:** 2019-10-22

**Authors:** Heather Wilson-Robles, Kelli Franks, Roy Pool, Tasha Miller

**Affiliations:** 660 Raymond Stotzer Pkwy, College Station, TX 77845 USA

**Keywords:** Osteosarcoma, Canine, Cell line, Xenograft

## Abstract

**Background:**

Canine and human osteosarcomas (OS) are notably similar and have a high rate of metastasis. There is a poor understanding of the tumor development process, predisposing causes, and varying levels of aggression among different cell lines. By characterizing newly developed canine osteosarcoma cell lines, treatments for people and pets can be developed. Of the seven subtypes of OS, three are represented in this group: osteoblastic (the most common), fibroblastic, and giant cell variant. To our knowledge, there are no other giant cell variant canine OS cell lines in the published literature and only one canine fibroblastic osteosarcoma cell line. Understanding the differences between the histologic subtypes in dogs will help to guide comparative research.

**Results:**

Alkaline phosphatase expression was ubiquitous in all cell lines tested and invasiveness was variable between the cell lines tested. Invasiveness and oxidative damage were not correlated with in vivo growth rates, where TOT grew the fastest and had the higher percentage of mice with metastatic lesions. TOL was determined to be the most chemo-resistant during cisplatin chemotherapy while TOM was the most chemo-sensitive.

**Conclusions:**

Further comparisons and studies using these cell lines may identify a variety of characteristics valuable for understanding the disease process and developing treatments for osteosarcoma in both species.

Some of this data was presented as a poster by KMF at the August 5th, 2017 National Veterinary Scholars Program in Bethesda, MA.

Characterization of 5 newly generated canine osteosarcoma cell lines.

Kelli Franks, Tasha Miller, Heather Wilson-Robles.

## Background

Immortalized cell lines are an invaluable resource and imperative to cancer research. However, the artificial conditions required to keep these cells alive in culture induce a variety of changes that likely would not occur in vivo [1–3]. The lack of a tumor microenvironment and tumor associated inflammation can alter surface marker expression as well as biologic pathways [1, 2]. These changes become more significant with the continued passaging of cells over time. Newly developed cell lines have undergone fewer passages and more closely resemble the primary tumor from which they were derived. These newly generated cell lines also have the potential to add much needed variety to in vitro research and the body of literature available for a given tumor type.

Osteosarcoma is the most common type of primary bone cancer that develops from osteoblasts and cells that form the osteoid matrix [4–9]. There are 1 to 3 cases per million reported annually in humans, and 70 to 75% of these cases affect people between the ages of 10 and 25 years [9]. In canine patients, OS represents nearly 85% of primary bone malignancies and affects over 8000 dogs per year [6, 7, 9, 10]. Osteosarcoma is more common in dogs, with some incidence reports as high as 27 times that of humans [9]. Middle aged or older dogs are usually affected though dogs less than 3 years of age represent a significant subset of those afflicted with this disease [6]. Additionally, a large dog breed predominance has been noted [9]. Canine and human OS are biologically and genetically similar, making canines a valuable representative model [11]. Similarities include a distinct male prevalence, large body size, primary tumor locations, unknown etiology of the disease process, and lung metastasis that is nearly always fatal [6, 11, 12]. Most cases do not present with metastasis as this develops later in the disease process [13]. The appendicular skeleton is most often affected in both canines and humans. Specifically, the metaphyseal area of long bones is the preferred site of the primary tumor [6]. Genetically these tumors have very similar gene expression patterns as well as biologic behavioral patterns [14–17]. Genome wide analysis confirms broad similarities in the mechanisms of progression and metastasis between species [16, 18].

Treatment for both species typically involves surgical resection and chemotherapy, with 60% of human patients reaching a 5 year survival time [9]. With metastasis present at diagnosis, survival time at 5 years drops to below 20% [9]. Distant metastasis occurs in over 80% of people treated with surgery alone [9]. In canine patients the expense of therapeutic treatment directs many clients to focus on pain management and analgesia [9]. There are few alternative treatments to chemotherapy because osteosarcoma is rare and has high genetic variability, making patient cohorts large enough to support biological studies difficult to achieve [19]. The average survival time in canines is 10–12 months with amputation and chemotherapy. Two-year survival rates are typically less than 20% [8, 20, 21]. New therapies are desperately needed for OS treatment and improvement of survival times.

The purpose of this manuscript is to describe five newly generated canine OS cell lines in an effort to better understand this devastating disease and allow for additional translational research to be performed in the future.

## Results

### Cell proliferation rates varied among the different cell lines

A 72-h proliferation assay was performed and yielded doubling times for each cell line. TOL, TOM, TOT and TOK were similar with doubling times of 35.7 h, 34.85 h, 33.78 h, and 32.07 h respectively. TOB had the fasted doubling time at 29.3 h. The previously immortalized and published canine OS cells lines, Abrams and BWKOS, had doubling times of 18.72 h and 17.02 h, respectively [13].

### ALP staining varied between cell lines

**Fig. 1:**
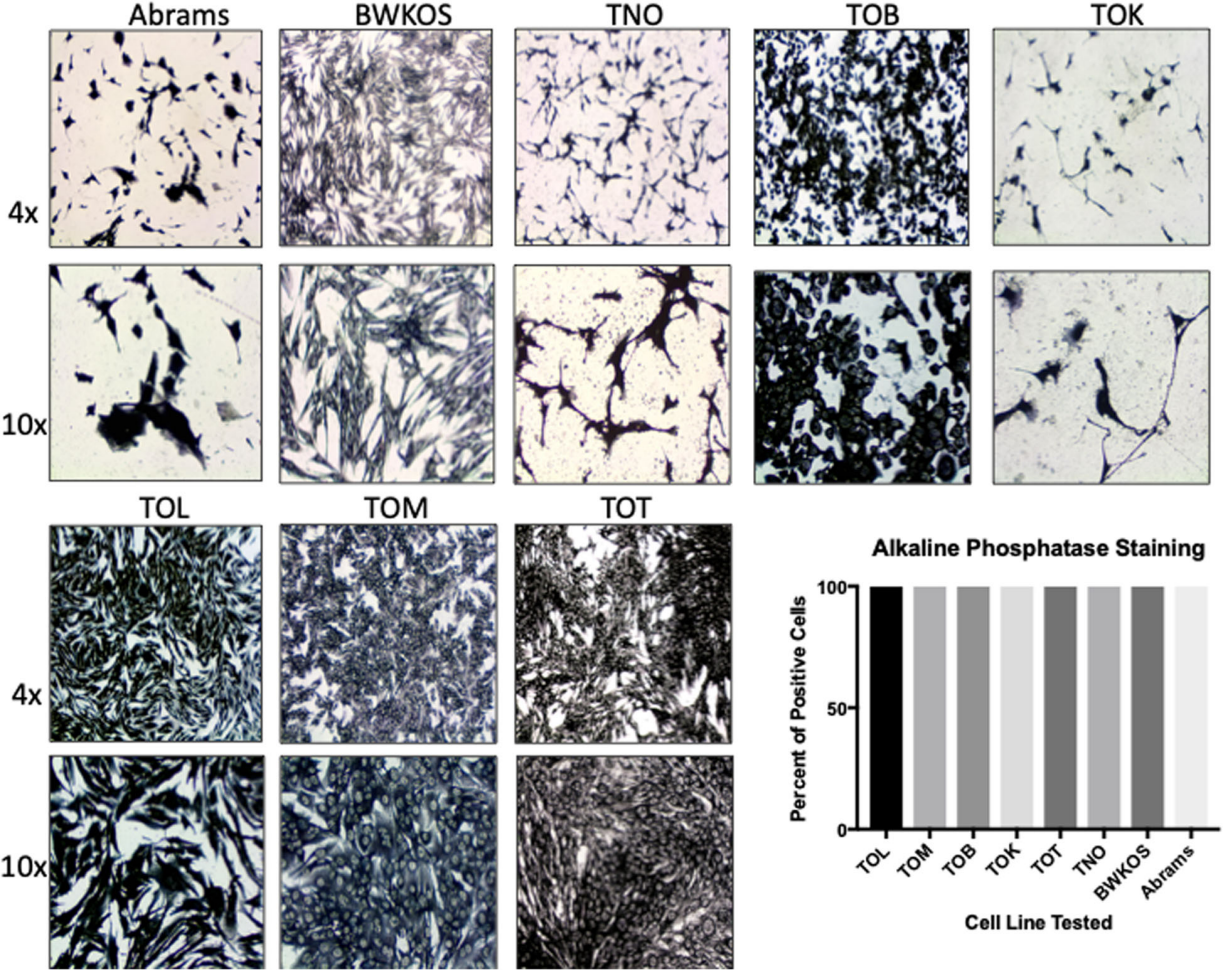
Alkaline phosphatase staining of Osteosarcoma Cell Lines. Alkaline phosphatase (ALP) staining. All cell lines were stained using a commercially available alkaline phosphatase kit. All cell lines, including the previously immortalized canine OS cell lines, Abrams and BWKOS, as well as the normal osteoblast cell line, TNO, stained positive for ALP in 100% of the cells. Variable staining was seen in each cell line with BWKOS and TOM having the lightest staining and Abrams and TOK having the darkest staining, similar to that seen in TNO

ALP staining performed on each cell line determined the percent of ALP positive cells present in each cell line. ALP expression was consistent between all cell lines as well as the normal osteoblastic cell line (TNO) and the two previously established OS cell lines, Abrams and BWKOS. All cell lines demonstrated 100% positive expression of alkaline phosphatase, though the degree of staining was variable between the cell lines (Fig. 1).

### Invasiveness also varied between cell lines

**Fig. 2:**
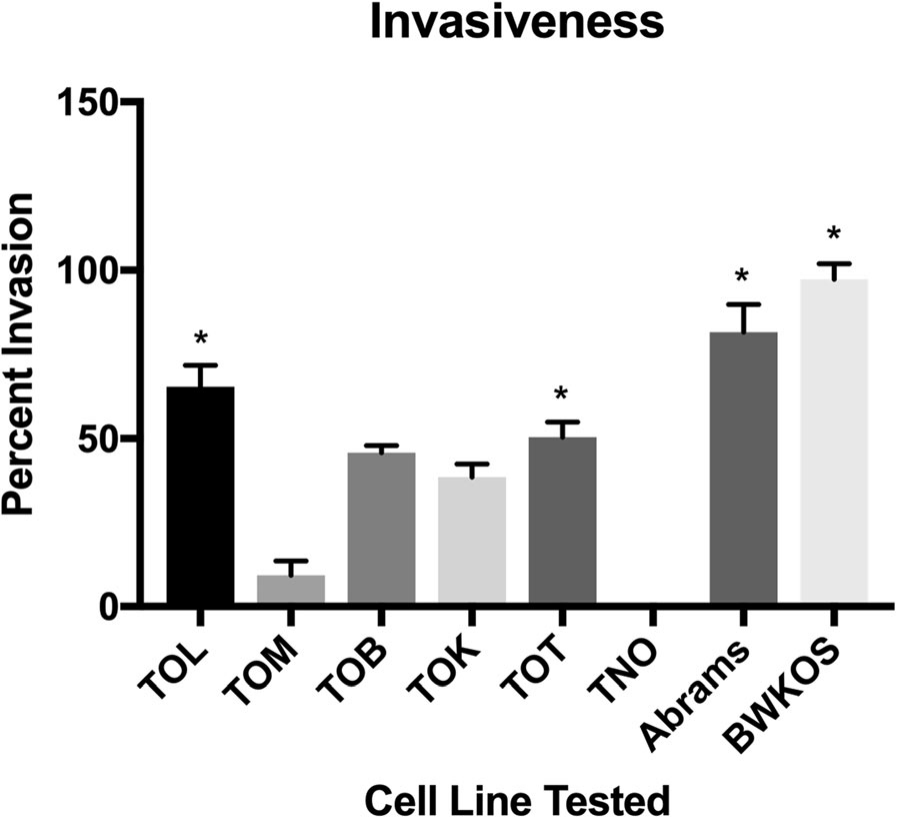
Invasiveness of Osteosarcoma Cell lines. Mean percent of cellular invasion through a matrigel basement membrane. The level of invasiveness varied widely between cell lines with TOL and TOT being the most invasive and TOM being the least invasive cell line of the newly generated cell lines. Abrams and BWKOS were the most invasive cell lines studied

Each cell line was measured for the percentage of cells that crossed the coating buffer, resembling a basement membrane, to get to the enriched media below. TOL had the highest percent of invasion with a mean of 65.33% (SD 6.31%) of cells invading through the membrane compared to controls. This was the closest cell line to the invasiveness found in both the previously established cell lines, Abrams (mean 81.5%, SD 8.36%) and BWKOS (Mean 97.3%, SD 4.61%). TOT had a mean of 50.42% (SD 4.58%) invasion followed by TOB with 45.86% (SD 2.13%) invasion through the basement membrane. TOK and TOM had the lowest mean percent invasion at 38.54% (SD 3.93%) and 9.26% (SD 4.3%), respectively. TNO was not invasive. Both the TOL (*p* = 0.0028) and TOT (*p* = 0.046) cell lines had significantly higher invasive ability when compared to the normal osteoblast cell line TNO, as did Abrams (*p* = 0.02) and BWKOS (p = 0.002) (Fig. 2).

### The degree of oxidative damage is higher in newly generated cell lines compared to existing immortalized cell lines

**Fig. 3:**
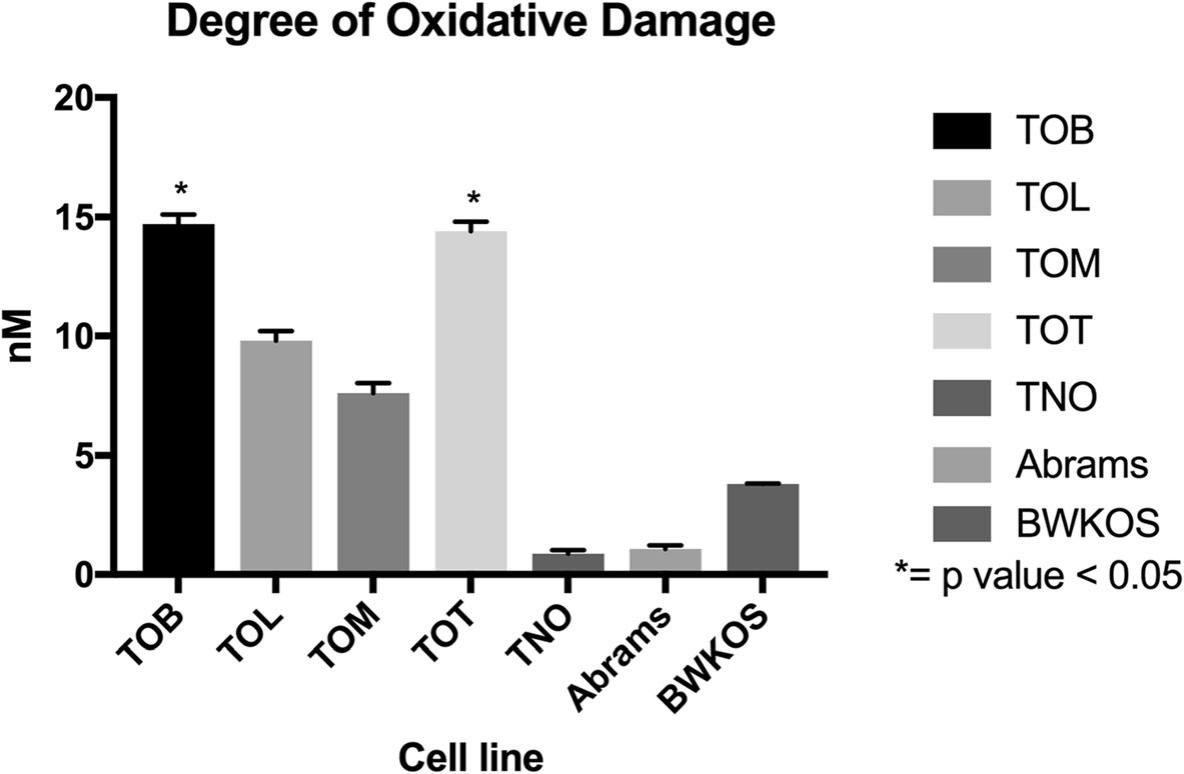
Degree of Inherent Oxidative Damage in Osteosarcoma Cell Lines. Mean concentration of 8-OHdG in nM in each cell line. The level of oxidative damage was highest in TOB and TOT and lowest in TOM. Oxidative damage was lowest in the established cell lines with Abrams being very similar to the normal osteoblast cell line TNO

The amount of oxidative damage inherently associated with each cell line was determined and compared to the TNO cell line as well as the previously established cell lines, Abrams and BWKOS. Exposure to mutagenic reactive oxygen species (ROS) has been implicated in cancer development and progression [22]. When exposed to ROS, 8-hydroxy-2′-deoxyguanosine (8-OHdG) is formed and can be measured as a sensitive indicator of physiologic and environmental DNA damage. The amount of 8-OHdG present in nM in each cell line was measured and recorded. TOB has the highest level of 8-OHdG with a mean of 14.74 nM (SD 0.4, *p* = 0.01). TOT had similar levels with a mean of 14.47 nM (SD 0.41, *p* = 0.02). TOL had a mean of 9.83 nM (SD 0.40, *p* = 0.4) and TOM had the lowest degree of oxidative damage with a mean of 7.61 nM (SD 0.42, p= > 0.99). Both TOB and TOT had a significantly higher degree of oxidative DNA damage when compared to the TNO cell line (mean 0.85 nM, SD 0.15). The previously established cell lines, Abrams and BWKOS, had low levels of inherent oxidative damage present. Abrams was very similar to TNO with a mean of 1.07 nM (SD 0.25) and BWKOS had a mean concentration level of 3.8 nM (SD 0.03) (Fig. 3).

### The five cell lines displayed variable sensitivity to cisplatin in vitro

Cisplatin is a platinum chemotherapy drug frequently used to treat osteosarcoma in both dogs and humans. Sensitivity to the drug was measured by determining cell viability after exposure to the drug at a variety of concentrations over several time points. Determination of IC50 revealed TOK to be the most chemo-resistant, with higher concentrations of cisplatin needed to inhibit growth and initiate cell death at 2 of the 3 time points measured. TOM and TOB are the two most chemo-sensitive with lower concentrations of cisplatin needed to induce cell death at all three time points. Indeed, an IC50 could not be determined for TOM at 72 h due to excessive cell death. The TOT cell line demonstrated initial resistance to cisplatin at the 24 h timepoint, however, the IC50 dropped significantly after 48 and 72 h of exposure to the drug. The previously established OS cell lines, Abrams and BWKOS, demonstrated similar sensitivities to cisplatin as the TOB and TOM cell lines. Table 1 lists all of the calculated IC50 data gathered for each cell line at each time point.

**Table 1:** IC50 data for all 5 cell lines after exposure to cisplatin (N/D = not determined)

	TOB (μM)	TOL (μM)	TOT (μM)	TOM (μM)	TOK (μM)	Abrams	BWKOS
IC50 (24 h)	15.91	64.96	95.52	20.26	213.30	52.46	13.57
IC50 (48 h)	12.05	41.94	16.58	6.53	12.25	8.58	3.977
IC50 (72 h)	6.91	23.23	4.85	N/D	64.30	10.02	50.73

### Three of the five cell lines are capable of enhanced adipocyte differentiation in vitro

**Fig. 4:**
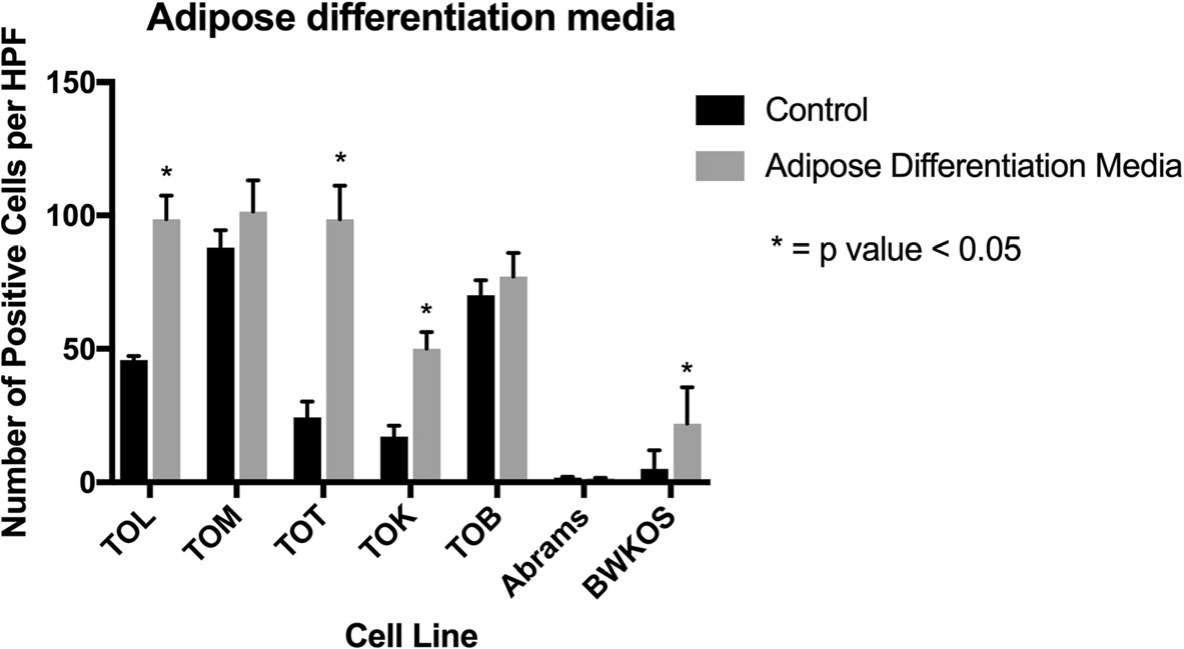
Degree of Adipocyte Differentiation in Osteosarcoma Cell lines. Three of the 5 cell lines were able to undergo adipocyte differentiation when exposed to ADM for 14 days. TOT, TOL and TOK had significant increases in the amount of intracellular lipid (*p* < 0.0001). BWKOS was capable of a small amount of adipocyte differentiation, though this was minimal compared to the newly generated cell lines

TOT, TOL and TOK were capable of enhanced adipocyte differentiation when grown in adipocyte differentiation media (ADM). In the TOT cell line, the control contained a mean of 24.11% cells staining positive with ORO (oil red O) compared to a mean of 92.5% positive cells on the ADM treated slides (*p* < 0.0001, SED 6.2). The TOL cell line had 44.64% positive cells in the control samples compared to 86.42% cells in the ADM treated samples (p < 0.0001, SED 3.9) and the TOK cell line had 17.2% positive cells in the control samples compared to 50% positive cells in the ADM treated cells (p < 0.0001, SED 3.4). The TOB and TOM cell lines had 70.2 and 87.13% positive cells in the controls versus 77.2% (*p* = 0.17, SED 4.7) and 96.4% (*p* = 0.056, SED 2.2) in the ADM treated samples, respectively. Abrams was incapable of adipocyte differentiation even with ADM media stimulation with a mean of 1.2% positive cells in the control and 0.8% in the ADM media (*p* = 0.47, SED 0.53). The BWKOS cell line was capable of some differentiation with 5% positive cells in the control versus 22% in the ADM media (*p* = 0.037, SED 6.84), however, this was minimal compared to the newly derived cell lines even though it was significant (Fig. 4 and Additional file 1: Figure S1).

### Three of the four cell lines tested were capable of enhanced osteogenic differentiation in vitro

**Fig. 5:**
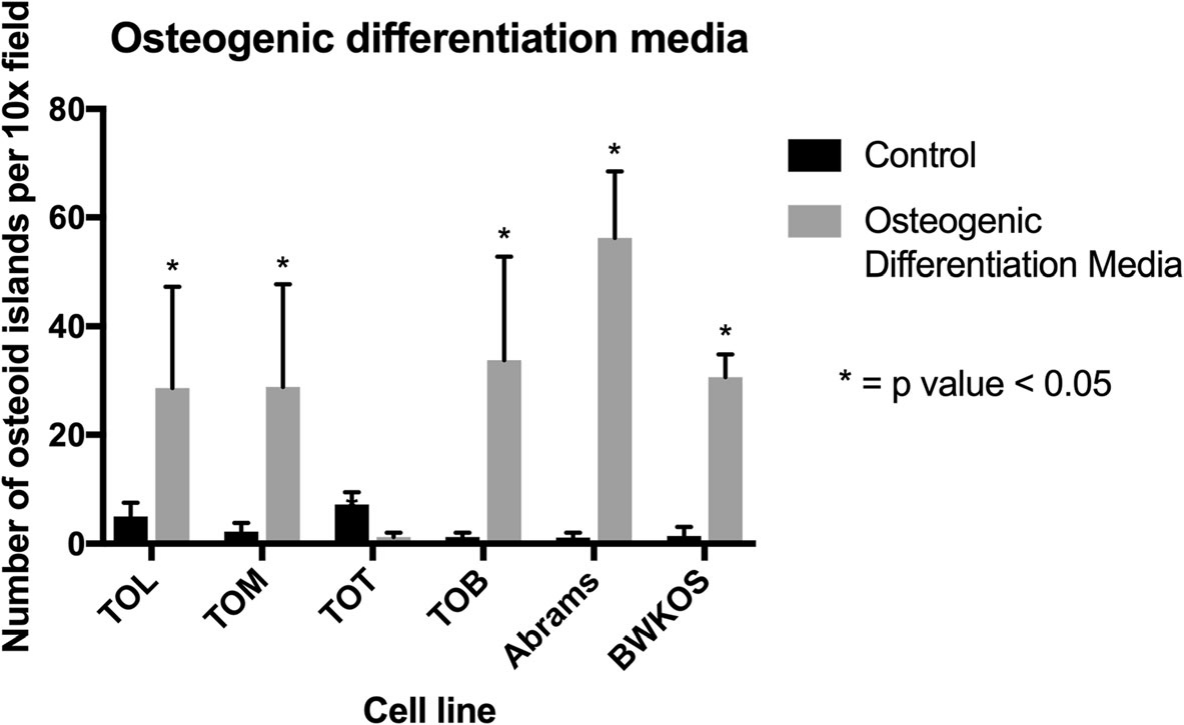
Degree of Osteogenic Differentiation in Osteosarcoma Cell Lines. Exposure to osteogenic differentiation media (ODM) demonstrated that 3 of 4 cell lines tested were able to produce a significantly higher number of bone islands than controls. The TOT cell line was surprisingly resistant to the ODM with a significant decrease in the ability to produce bone islands after exposure to ODM. Abrams exceeded the other cell lines in its ability to produce osteoid islands.

TOL, TOM and TOB were capable of creating a significantly higher number of osteoid islands in vitro when exposed to osteogenic differentiation media (ODM). The control sample for TOL had a mean of 5 osteoid islands per 10x field compared to 28.6 osteoid islands in the ODM sample (*p* = 0.023, SED = 8.43). For the TOM cell line there was a mean of 2.2 osteoid islands per 10x field compared to 28.8 in the ODM sample (*p* = 0.014, SED = 8.5), and for TOB there was a mean of 1.2 osteoid islands per 10x fields whereas the ODM sample had a mean of 33.8 osteoid islands per 10x field (*p* = 0.005, SED = 8.47). The TOT cell line was surprisingly resistant to ODM with a significantly larger number of osteoid islands in the control (mean = 7.2) compared to the ODM sample (mean = 1.2, *p* = 0.0005, SED = 1.086). The two previously established canine OS cell lines, Abrams and BWKOS, were highly capable of creating osteoid islands. Abrams generated very large osteoid islands with nearly every cell staining positive for cytoplasmic osteoid. For the Abrams cell line there was a mean of 1 osteoid islands per 10x field compared to 56.2 in the ODM treated cells (*p* < 0.0001, SED 5.53). BWKOS has very similar osteoid island production to that seen with TOL, TOM and TOB (Fig. 5 and Additional file 1: Figure S1) with a mean of 1.4 osteoid islands in the control versus 30.6 in the ODM treated cells (*p* < 0.0001, SD 2.03).

### Only one of the 4 cell lines exposed to chondroblastic differentiation media (CDM) was capable of chondrogenic differentiation

**Fig. 6:**
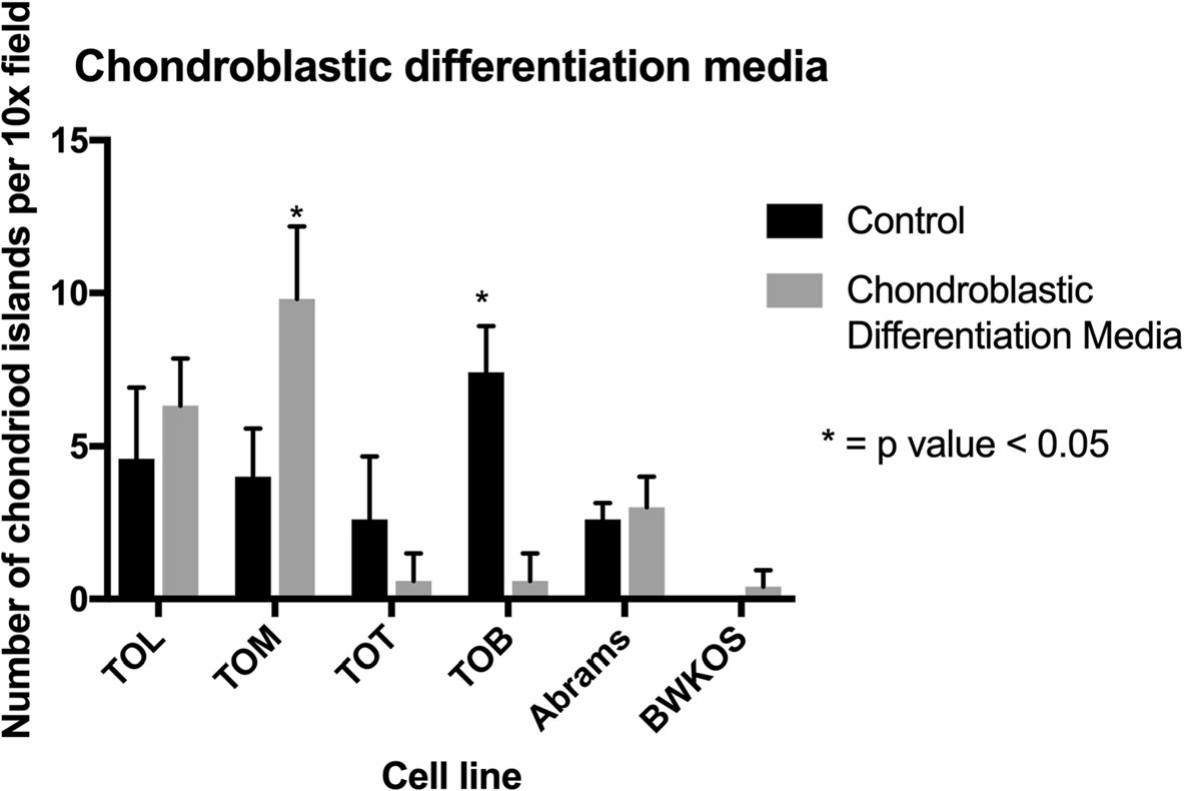
Degree of Chondroblastic Differentiation in Osteosarcoma Cell Lines. Exposure to chondrogenic differentiation media (CDM) demonstrated that only one of the four cell lines tested was capable of chondrogenic differentiation. The TOM cell line produced significantly more chondroid islands compared to controls after exposure to CDM. TOB had a significantly higher number of chondroid islands in the controls than the CDM media. Neither Abrams nor BWKOS were capable of generating chondroid islands

The TOM cell line was the only one of the four cell lines tested capable of significantly producing more chondroid islands after exposure to CDM (mean- 9.8 per 10x field) compared to controls (mean-4 per 10x field, *p* = 0.002, SED = 1.28). The TOB cell line was surprisingly resistant to the CDM producing significantly fewer chondroid islands (mean- 0.6 per 10x field) compared to controls (mean- 7.4 per 10x field, p < 0.0001, SED = 0.78). The TOT line again produced fewer chondroid islands in the CDM media (mean-0.6) compared to controls (mean-2.6, *p* = 0.08, SED 1.98). The TOL line produce slightly more chondroid islands in the CDM (mean- 6.3) compared to the controls (mean- 4.6, *p* = 0.29, SED 1.51) but this was not a significantly higher amount. Neither of the previously established canine OS cell lines was capable of any significant chondroid production with a mean number of 2.6 chondroid islands per 10x field in the control versus 3 chondriod islands in the CDM media (*p* = 0.45, SED 0.51) for the Abrams cell line and 0 chondroid islands for the control versus 0.4 chondroid islands in the CDM media (*p* = 0.14, SED 0.24) (Fig. 6 and Additional file 1: Figure S1).

### Four of the five cell lines studied were able to reliably produce tumors in vivo

**Fig. 7:**
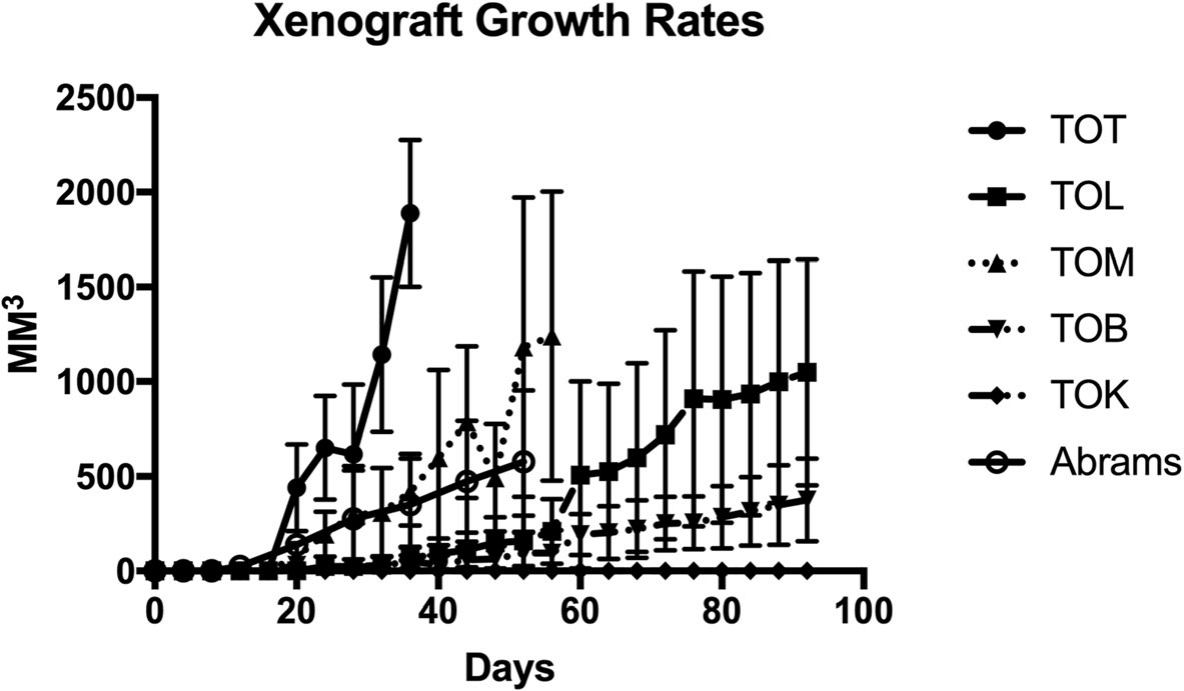
Xenograft Growth Rates for Osteosarcoma Cell Lines. Tumor growth rates over a 12 week period. TOT xenograft reached 2 cm in 5 weeks. This indicates a more aggressive tumor behavior

All mice involved started the experiment in good health and body condition with an average weight of 25.6 g. In vivo*,* TOT was the most aggressive of the 5 cell lines studied. Xenografts from TOT reached 2 cm in less than 36 days in all 6 mice injected (mean tumor volume 1889 mm^3^, SD 387.6). Several, though not all, of the TOM xenografts also demonstrated a rapid growth rate compared to the other cell lines with large tumors necessitating euthanasia in all 6 mice by day 56 (mean tumor volume 1241.33 mm^3^, SD 762.77). Five of the 6 mice developed tumors in each of the TOL (mean tumor volume 1048.3 mm^3^, SD 595.15) and TOB (mean tumor volume 375.0 mm^3^, SD 219.93) groups and were euthanized due to ulcerations of the masses on day 84 after injection. None of the 6 mice injected with the TOK cell line were able to develop tumors after 12 weeks of monitoring. The Abrams cell line was also injected into 6 mice and growth rates recorded for 52 days (mean tumor volume 578.8 mm^3^, SD 376.36). This cell line produced tumors in all 6 mice and had a similar growth rate to the TOM cell line (Fig. 7).

### Histologically, xenografts compared favorably with the primary tumors from which they were derived

**Fig. 8:**
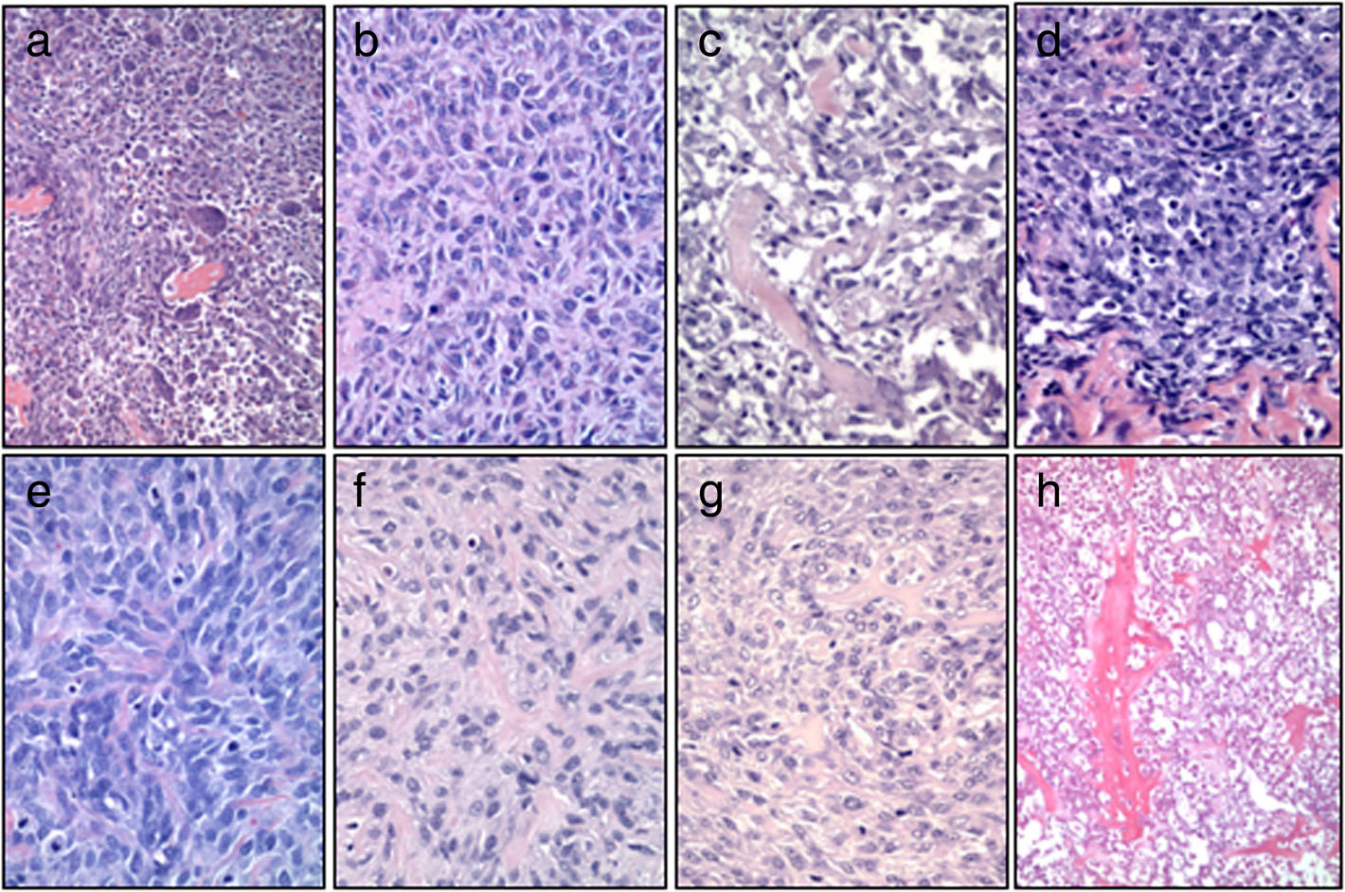
Histopathologic Comparison of Primary Osteosarcomas from Canines and Xenografts. Example **h**&**e** images of the primary tumor from which the cell lines were derived. **d**-**f**&**h.** Example **h**&**e** images of the xenografts grown in athymic nude mice. **a** and **e**- TOT; **b** and **f**- TOM, **c** and **g**- TOL, **d** and **h**- TOB

Original haemotoxylin and eosin (H&E) stained slides from 4 of the 5 cases were compared to H&E stained slides of the murine xenografts generated from each cell line. TOK did not produce tumors in mice so there was no tissue available for comparison. Additionally, slides from the primary tumor used to generate Abrams were not available to us for comparison. Histologic comparisons were made by an osteopathologist (RP). In general, the histologic characteristics for the tumors were preserved in vivo (Fig. 8)*.* For the TOT cell line three of the four tumor histological patterns present in the original tumor (Fig. 8 a) were present in the xenograft (Fig. 8 e). A fusiform to spindle cell pattern, compact polygonal cell pattern of cells with tiny slit-like intercellular spaces somewhat resembling the pattern in some squamous cell carcinomas, and ovoid multinucleated tumor cells with lesser numbers of spindle cells were seen in both the primary tumor and the xenografts. However, a mixed pattern of spindle cells bordered by polygonal and ovoid cells with a few multinucleated giant cells was not seen in the xenografts. Additionally, while tumor bone formation was present in the original tumor tissue from the proximal humerus, no tumor osteoid was present the xenografts. The mitotic index (MI) in the primary tumor was 30 (3 mitoses per 40x field). In the xenografts the MI was significantly higher ranging from 50 to 90 depending on the murine xenograft evaluated.

The TOM xenografts (Fig. 8 f) retained the two of the three major cellular patterns composed of a moderately dense population of mixed spindle cells and random areas of sheets of highly cellular tumor. However, like TOT, these xenografts also lacked any tumor bone formation or hyperchromatic tumor cells with angular profiles resembling osteoblasts seen in the primary tumor (Fig. 8 b). The MI in the primary tumor approached 50 in some areas however the xenografts consistently had a lower MI in the evaulated murine xenografts ranging from 2 to 30.

The tumor from which TOL was derived consisted of three major cellular patterns including a meshwork of hyperchromatic polygonal cells of moderate cell density containing small numbers of tiny spicules of tumor with angular profiles, a moderately dense filigree of small and larger haphazard spicules of tumor bone some of which is mineralized, and random sheets of highly cellular tumor containing a few spicules of tumor bone in which tumor cells were loosely packed but had tiny slit like spaces between the hyperchromatic tumor cells having angular profiles (Fig. 8 c). The TOL xenografts (Fig. 8 g) contained small irregularly shaped deposits of tumor bone produced by hyperchromatic spindle tumor cells that are set in an indistinct fibrillar matrix and retained the three major cellular patterns. The MI in the primary tumor was less than 1; however the MI in the xenografts was much higher at 30.

The TOB cell line was derived from a metastatic lesion (Fig. 8 h) collected at euthanasia from a fibroblastic osteosarcoma. Slides from both the primary and metastatic lesions were available for comparison. The primary neoplasm was composed of haphazard streams, rudimentary bundles, and fine threads of variably pleomorphic spindle cells within a largely collagenous matrix that was multifocally punctated by areas of neoplastic cells entrapped within osteoid or forming woven, partially mineralized bone spicules. The metastatic lesion (arising from the sternum) had undergone severe coagulative necrosis before collection at necropsy, however, appeared to have very similar features to the primary tumor. The xenografts from the TOB cell line (Fig. 8 d) produced the most bone of all of the cell lines studied with a central nodule of tumor bone formation created by hyperchromatic tumor cells having angular profiles resembling osteoblasts. There were random peripheral areas of spindle cells and deeper peripheral areas of the xenograft had haphazardly arranged hyperchromatic polygonal and spindle cells. The MI of the primary tumor was 25 while the xenograft had a MI of 50.

### Three of the 5 murine xenograft models studied developed pulmonary metastases

**Fig. 9:**
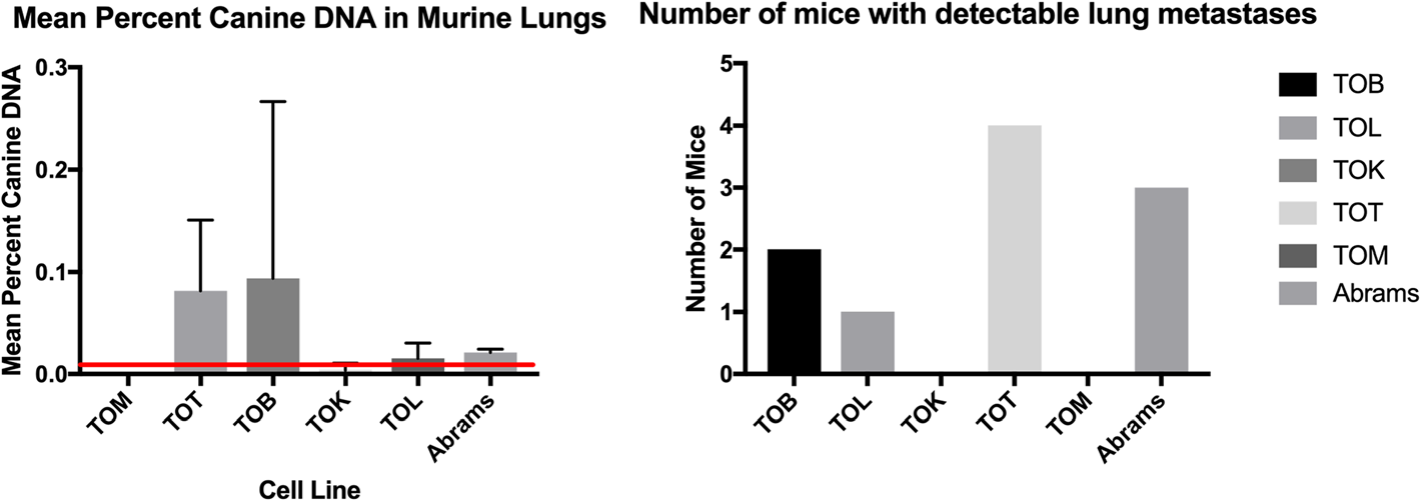
Metastatic Rate for Osteosarcoma Cell Lines in a Murine Xenograft Model. Left. Mean percent canine DNA in lungs of 2 of the canine OS cell lines. Micrometastasis is detected using canine specific primers to quanitify the amount of canine DNA in the lungs of each mouse. Four of the 6 mice in each group were used to calculate the means. The minimum detection level of the assay is 0.02% as demonstrated by the red line. Right- Number of mice in each group with detectable metastases. Three of the 5 cell lines had mice with detectable levels of foreign canine DNA present in murine lung samples

Whole lung quantitative DNA analysis was performed on 4 of the 6 mice in each group. Species specific primers were used to identify foreign canine DNA in the lungs of the mice. This assay can predictably detect as little as 0.02% foreign DNA in samples. The TOB (2 of 4 mice), TOL (1 of 4 mice) and TOT (4 of 4) cell lines each had evidence of micrometastatic lesions present in lung samples. The amount of canine DNA was highest in the TOT xenograft mice. The mean percent of DNA in the TOM and TOK lung samples were 0 (SD 0) and 0.003 (SD 0.007), respectively. TOB had the highest mean percent canine DNA in the lung samples with 0.094% (SD 0.17). TOT has a mean percent of canine DNA of 0.082% (SD 0.068) and TOL had a mean percent of 0.015% (SD 0.01) with only one mouse registering above the 0.02% minimum reliable detection level of the assay. For the Abrams cell line the mean percent of canine DNA detected was 0.021% (SD 0.003) with 3 of the 4 mice having means above the 0.02% cut off (Fig. 9).

## Discussion

Each of the five cell lines generated and characterized here demonstrated slightly different biologic behavior. To the authors’ knowledge this is first description of the characteristics of a canine giant cell variant osteosarcoma (TOT) and only the second of a canine fibroblastic osteosarcoma (TOB) in the literature [23]. These two histologic subtypes represented two of the most aggressive cell lines evaluated in vivo in this group.

Some of the more interesting findings in the characterization of these cell lines include the longer doubling times of the new cell lines compared to the existing cell lines, the difference in the degree oxidative in the newly generated cell lines compared to Abrams and BWKOS and the degree of plasticity in the newly generated cell lines compared to the more established cell lines.

Newly generated cell lines often take time to immortalize and “learn” to grow on plastic in flasks. For this reason, it is not unexpected that the new cell lines require a little more time to divide and grow to confluence within the flask. It is expected that over time these cell lines, especially the more aggressive ones, will shorten their doubling times to be more in line with those of Abrams and BWKOS.

The increased degree of oxidative damage present in the new cell lines as compared to the established cell lines was unexpected. Cancer cells and cell lines have been shown to have decreased levels of antioxidant enzymes such as manganese superoxide dismutase (MnSOD), leading to higher levels of ROS and increased DNA damage [24]. This damage further enhances the genetic instability of these cells allowing them to procure additional survival advantages. As these new cell lines adjust to cell culture, they may have enhanced oxidative stress contributing to this unexpected difference.

The previously established cell lines were only capable of differentiating when exposed to the ODM (Abrams and BWKOS) and ADM (BWKOS). This lack of plasticity was unexpected for these established cell lines. The newly generated cell lines demonstrated an increased ability to differentiate along adipocyte lines, however, there was a fair amount of lipid present within the cytoplasm of the cells even within the control cells. Altered metabolism is an emerging hallmark of cancer. Cancer cells are known for their ability to obtain energy from glycosis even with ample amount of oxygen present. This is referred to as the Warburg effect. This metabolic pathway causes cancer cells to increase the generation of cytoplasmic lipid droplets in the same way that cells with reduced access to nutrients and lipids would [25, 26]. This may explain why many of the cells had moderate amounts of lipid within them from the start and why they were able to produce significant amounts of lipid when stimulated to do so because the machinery within the cell was already turned on.

The TOT cell line consistently demonstrated an aggressive biologic behavior. This cell line had a high level of invasiveness, a high degree of oxidative damage, a high propensity for adipocyte plasticity, a rapid growth rate in a murine model with 100% xenograft formation and 100% metastatic rate. The cell line also had the highest MI in the xenografts examined. This aggressiveness mirrors the clinical picture associated with the primary tumor from which it was derived. This patient was treated aggressively with amputation of the affected limb and carboplatin chemotherapy. However, the progression free interval (PFI) was only 183 days when the patient presented with bone metastasis and the overall survival time (OST) was only 233 days.

The TOB cell line had high invasive capabilities, a relatively quick doubling time, high oxidative damage, cisplatin sensitivity, very little plasticity, slow growth in vivo*,* but a moderate metastatic rate. This was the only cell line derived from a metastatic lesion and this patient has the longest clinical course of the 5 dogs represented in this study. This patient, also treated with amputation and carboplatin chemotherapy, had a PFI of nearly 7 years (2383 days) with the presentation of lung and sternebra metastasis and an OST of 2402 days. Histologically, this tumor produced the most bone in the murine studies and had a moderately high MI. The characteristics of this cell line are not universally aggressive and the ability of this cell line to invade and spread may be sequelae of the fact that it was derived from a metastatic lesion rather than the true aggressive nature of the primary tumor.

The TOL cell line had many similarities to the TOT cell line in that it had the highest invasive ability, a moderate amount of oxidative DNA damage and plasticity, moderately high in vivo and in vitro growth rates and was fairly chemotherapy resistant. The metastatic rate for this cell line was low with only 1 in 4 mice demonstrating measurable metastatic disease however, if the mice had been allowed to live longer with their tumors this may have increased. The MI in the primary tumor was low but the xenografts had a moderately high MI of 30. The clinical course of this patient was altered by a surgical complication in which the dog developed a multi-drug resistant bacterial infection at the amputation site and was only able to receive 1 dose of chemotherapy before a significant treatment delay led to the development of pulmonary metastatic disease. The PFI for this patient was 130 days with an OST of 232 days.

Despite an aggressive clinical course for the TOM cell line and the relative sensitivity to cisplatin seen in vitro this patient developed lung metastases 139 days after diagnosis even though she was treated with amputation and carboplatin chemotherapy. TOM was the most plastic of the cell lines evaluated with the ability to form exaggerated levels of adipocytes, bone islands and chondroid when stimulated to do so. The OST for this patient was 215 days even with additional chemotherapy. The TOM cell line represented one of the least biologically aggressive cell lines of those studied here and perhaps this patient would have benefited from cisplatin therapy rather than carboplatin.

TOK cell line was the least aggressive cell line studied. Unfortunately, this cell line developed contamination during the study that could not be eliminated, and the cell line died out before completion of the oxidative damage assay as well as the osteogenic and chondrogenic differentiation assays could be done. This cell line had moderate ALP staining, moderate invasive characteristics and moderate plasticity with ADM. This tumor did not form any tumors in mice and no metastatic lesions were detected. It was, however, the most drug resistant cell line studied. This tumor displayed a fairly typical clinical behavior as the patient developed metastatic lesions at 286 days and lived a total of 380 days after diagnosis. This patient was treated with amputation and adjuvant carboplatin chemotherapy.

## Conclusion

Each of the 5 OS cell lines studied here display a variety of characteristics. There is a wide variety of biologic behaviors seen between the different cell lines with varying levels of invasiveness, ALP expression, and proliferation rates. The tumors in vivo varied widely in metastatic potential and growth rates as well. The cell lines also maintained many of the original histologic characteristics in the xenografts that were seen in the primary tumors. The three histological subtypes studied will broaden therapeutic treatment testing and provide new perspectives for understanding the complex process of tumor development and metastasis.

## Methods

### Cell culture

Canine osteosarcoma (OS) cell lines TOB, TOL, TOK, TOM, and TOT were generated with owner permission from patients seen at the Texas A&M Small Animal Clinic Oncology Department in College Station, Texas (AUP 2015–0350 CA) (Table 2). Samples were collected from primary or metastatic sites either after amputation or euthanasia in all cases. A normal osteoblast cell line (TNO) was also generated from a patient after euthanasia and used for comparison. The Abrams cell lines was generously provided by Dr. David Vail at the University of Madison Wisconsin and the BWKOS cell line was graciously provided by Mrs. Christina Mazcko at the National Cancer Institute Comparative Oncology Trials Consortium. All cell lines were grown in T75 and T175 flasks with RPMI 1640 media supplemented with 10% fetal bovine serum, 1% penicillin, 1% streptomycin, and 1% Fungizone. Cells were maintained in an incubator at 37 °C with 4% CO_2_.

**Table 2:** Osteosarcoma cell line origins

Cell Line Name	Signalment	Location	Diagnosis
TOB (metastatic lesion)	5 y/o FS Neapolitan Mastiff	Right distal tibia (1°); 5th sternebra (2°)	Fibroblastic OS
TOK (primary tumor)	7 y/o MN Golden Retriever	Left proximal humerus	Osteoblastic OS
TOL (primary tumor)	4 y/o MN St. Bernard	Right distal radius	Osteoblastic OS
TOM (primary tumor)	12 y/o FS Australian Cattle Dog	Left proximal tibia	Osteoblastic OS
TOT (primary tumor)	10 y/o FS Greyhound	Right proximal humerus	Giant Cell Variant OS
TNO (normal osteoblasts)	7 y/o FS Labrador Retriever	Right radius	N/A

### Alkaline phosphatase staining

Cells were grown in flasks as described above to 75% confluence. They were trypsonized, collected and washed in PBS. 6 × 10^4^ cells were counted and plated on chamber slides (LAB-TEK, Thermo Fisher Scientific). One milliliter of MSC growth medium was added to each slide. Slides were incubated at 37 °C in 5% CO2 until 75–80% confluence was achieved. The media was removed, and the slides were fixed with acetone and washed for 5 min in tap water The ImmPRESS AP Polymer kit was used to stain the cells for alkaline phosphatase (Vector Laboratories, cat # MP5402) according to the manufacturer’s instructions. Briefly the cells were washed in buffer for 5 min before they were incubated in 2.5% normal horse serum as a blocking agent. The cells were incubated with a primary mouse antibody diluted 1:50 in buffer. The cells were washed again for 5 min in buffer and incubated with ImmPRESS-AP polymer Reagent and washed 2x’s in buffer before the alkaline phosphatase substrate was added for 24 h. The cells were rinsed in tap water and evaluated under the microscope.

### Invasion assay

Coating buffer was made from 0.7% NaCl and 0.1 M tris in distilled water. It was filtered using a 0.2 μm sterile syringe filter and stored at 4 °C. Matrigel Matrix (Corning, Tewksbury, MA, USA) was stored at 4 °C. The coating solution was made for a final concentration of 250 μg/mL. Fifty-four μL of matrigel was mixed into 1946 μL of coating buffer. The experimental, positive control and negative control wells were completed in triplicate and designated + 1, + 2, and 0 respectively. Three thinserts (Greiner Bio-one, Kremsmünster, Austria) per cell line were placed in a 24 well plate (VWR, St. Louis, MO, USA). These were coated in 100 μL of coating solution. The thinserts were placed in the incubator at 37 °C for 2 h. A cell suspension was prepared with 2 × 10^6^ cells in 3 mL PBS and was added to each tube. The solution was centrifuged for 3 min at 1300 RPM. After decanting the supernatant, 2 mL of serum free media was added to the cell pellet and mixed with a pipette. On the 24 well plate, 600 μL of serum free media was placed in wells marked 0. Six hundred microliters of media containing serum was placed in wells marked + 1 and + 2. Thinserts were placed in wells designated 0 and + 2, while the coated thinserts were placed in the + 1 wells. 200 μL of the cells in serum free media were added to the top of the thinserts. The 24 well plate was placed in the incubator for 20 h at 37 °C. With a new 24 well plate, 450 μL of serum free media with 8 μM calcein-AM was added to each well. The thinserts were moved to the new plate containing the calcein-AM solution. The plate was incubated for 45 min at 37 °C. Each thinsert was removed and placed in a new well of a 24 well plate. The thinsert was aspirated and material on top of the thinsert was discarded. On a new 24 well plate 500 μL of trypsin-edta was added to each well. The thinserts were placed into the trypsin-edta wells. This plate was incubated for 15 min at 37 °C. Thinserts were discarded. The cells and trypsin were mixed and 200 μL were pipetted into new wells of a black 96 well plate in duplicate. The plate was read by the fluorescent plate reader (excitation 485 nm/emission 520 nm) (BioTek Synergy 2, Winooski, VT, USA).

Invasion index percentage was calculated using the formula:

Invasion index % = (experimental average – negative control average)/(positive control average – negative control average) × 100%.

### Cell proliferation

A white 96 well plate was labeled in triplicate with the cell line and number of cells plated. Initially, a variety of cell concentrations ranging from 500 to 3000 cells per well were plated to determine the best linear growth rate concentration. The appropriate amount of media was added to cell samples to reach a concentration of 100 μL needed per well. The plate was incubated for 2.5 h at 37 °C. Ten μL of the substrate and 10 μL of enzyme from the RealTime Glo Viability Assay (Promega, Madison, WI, USA) were mixed into 4980 μL of media to make a 2X solution. The solution was placed into a hot water bath for 5 min. Media was removed from the 96 well plate and 50 μL of new media was added to wells. Fifty microliters of substrate-enzyme solution was added to each well. The plate was incubated for 1 h at 37 °C. The Biotek Synergy 2 plate reader was prewarmed to 37 °C before the first reading. The plate was read on the prewarmed fluorescent plate reader (Biotek Synergy 2, Winooski, VT, USA) at 24, 48, and 72 h (488 nm) post plating. Results were graphed to determine linearity of concentrations. The following formula was used to determine the doubling time for each cell line:

(t2 - t1)/3.32 x (log n2 - log n1) where t is time and n number of cells (27) {Hayflick, 1973 #8}.

### Cisplatin sensitivity

Cisplatin was received as a 1 mg/mL solution at 3.333 mM (Professional Compounding Centers of America, College Station, TX). From this a 400 μM solution was prepared. For the serial dilution of 200 μM, 100 μM, 50 μM, 25 μM, 12.5 μM, 6.25 μM, 3.125 μM and 0 μM concentrations were generated. An acrid orange/ propidium iodide (AO/PI) stain was used to count live and dead cells per well for all cell lines. These surviving cell count numbers were determined to be linear. Media was added to cell volume to reach appropriate concentrations for 100 μL of cell solution per well. A white 96 well plate was used and all experiments were done in triplicate. The plate was incubated for 2.5 h at 37 °*C. media* was removed from the plate and the various serially diluted cisplatin concentrations in media were added to the wells in triplicates. 10 μL of MT Cell Viability substrate and NanoLuc Enzyme (Promega, Madison, WI, USA) were added to 4980 μL media and the solution was placed in the hot water bath for 5 min. 50 μL of the enzyme-substrate solution was added to all wells. The plate was incubated for 1 h at 37 °C and read on a prewarmed BioTek synergy 2 at excitation 485 nm/emission 520 nm. The plate was incubated at 37 °C before being read at 24, 48, and 72 h post plating.

### Differentiation media assays

Chondrogenic, osteogenic, and adipogenic differentiation media assays were performed. For the osteogenic and adipogenic assays, 6 × 10^4^ cells were counted and plated on chamber slides (LAB-TEK, Thermo Fisher Scientific). One milliliter of MSC growth medium was added to each slide. For the chondrogenic assay, 1 × 10^5^ cells were plated on chamber slides. One milliliter of MSC growth medium was added to each slide. Slides were incubated at 37 °C in 5% CO2. When the chondrogenic plate samples grew spheres at 60 h post plating, media was removed. Two slides received 1 mL of chondrogenic differentiation media and two slides received 1 mL of MSC growth medium. For the adipogenic assay cells reached 80–90% confluency before media was removed. Two slides received MSC growth media and two slides received adipogenic differentiation media. For the osteogenic assay cells reached 100% confluency before removing media and adding osteogenic differentiation media to two slides and MSC growth media to two slides. All differentiation slides were incubated at 37 °C. Every three days the media was changed. The chondrogenic and osteogenic slides were incubated for 21 days and the adipogenic slides was incubated for 14 days. Differentiation of cell lines was determined by doing a 500-cell count differential per slide of cells grown in the various differentiation media and cells grown in standard media as a control.

### Oxidative damage

The Trevigen HT 8-oxo-dG ELISA kit II (Gaithersburg, MD) was purchased to assess the level of inherent oxidative DNA damage in each of the cell lines. The assay was completed according to the manufacturer’s instructions. Briefly, cells from each cell line were grown to confluence in T75 flasks (Corning), washed, trypsonized and collected. Cells were diluted in ice cold PBS at 1 × 10^6^ cells/mL. DNA was extracted using the manufacturer’s protocol and quantitated spectrophotometrically (1OD_260_ = 50 μg/mL) to a final concentration between 200 and 1000 μg/mL. 100x cations, provided by the kit, were added to the DNA solution for final 1x concentration. The 8-OHdG standards and cell mixtures were added to a 96 well plate. Twenty-five microliters of the murine anti 8-OHdG monoclonal antibody solution (diluted 1:250) were added to each well (25 μL of diluent only were added to blank wells for negative controls) and thoroughly mixed. The plate was sealed and incubated at 25 °C for 1 h. Wells were washed 4 times with PBST and 50 μL of the goat anti-mouse IgG-HRP secondary antibody solution (diluted 1:500) was added to each well. The plate was again sealed and incubated at 25 °C for 1 h. The plate was washed again with PBST and 50 μL of pre-warmed TACS-Sapphire colorimetric substrate (provided by the kit) was added to all wells and the plate was incubated in the dark at 25 °C for 15 min. Fifty microliters of 0.2 M Hcl was added to each well to stop the reaction and absorbance was read immediately at 450 nm on a BioTek Synergy 2 fluorescent plate reader (Winooski, VT, USA). The amount of oxidative damage present in each cell line was calculated using a standard curve generated with Graphpad Prism 7 software.

### Growth and metastasis

A total of 30 female athymic nude mice were purchased from Envigo for the purposes of this study. All animal experiments were approved by the Texas A&M University Intuitional Animal Care and Use Committee (IACUC 2017–0127). Mice were housed in sterile cages with sterile bedding and 2–4 mice per cage. Mice were fed a sterile irradiated mouse chow. Mice were handled using sterile PPE under a sterile hood. Six mice were assigned per group in 5 groups by cell line. Media was removed from cell cultures and the T175 flask was rinsed with 5 mL PBS. The flasks were trypsonized and incubated for 12 min at 37 °C. Tuberculin syringes with 27-gauge needles were chilled in ice. A cell suspension of 1 × 10^6^ cells in 170 μL Corning matrigel matrix was injected subcutaneously in the right flank. The animal’s weights were recorded, and the ears were notched for identification purposes. Every 2 to 3 days after injection, weight and tumor size were measured and recorded. When tumors reached 2 cm in diameter, became ulcerated, or when the mouse lost up to 20% of its body weight, they were humanely euthanized. Otherwise mice were followed for tumor growth for 10 weeks. Mice were euthanized in accordance with AVMA guidelines using CO_2_ inhalation provided by a compressed CO_2_ gas cylinder at a rate of 2–3 l per minute in a sealed 10 Liter volume chamber until they were unconscious (CO_2_ narcosis) followed by cervical dislocation. Tumors as well as whole lung tissue was collected from each mouse after euthanasia. For each cell line, tissues were collected and stored in Invitrogen RNAlater, formalin or flash frozen in liquid nitrogen. After 2 days in formalin all tissues were removed, rinsed and placed in 70% ethanol.

### Whole lung DNA analysis

DNA was isolated from lung tissue using DNeasy® Blood & Tissue kit (Qiagen, Chatsworth, CA) in accordance with the manufacturer’s protocols. Quantitative PCR was performed using equal amounts of DNA with biological and technical triplicates (SsoAdvanced™ Universal SYBR® Green Supermix and CFX Connect™ Real-Time PCR Detection System, BioRad, USA). The primers are listed in Table 3. PCR conditions for human and canine primers were 40 cycles of denaturation at 95 °C for 10 s, annealing at 57 °C for 30 s, and chain extension at 65 °C for 5 s. The relative fold change between control and tumor- bearing mice, standard error, and statistical significance via a Pair Wise Fixed Reallocation Randomization Test© was calculated using REST© software (Pfaffl et al. 2002). A standard curve was created using known amounts of canine DNA in normal mouse lung DNA. This curve was used to interpolate the percentage of foreign species (i.e. canine) DNA present in each sample.

**Table 3:** Primer pairs used in qPCR

Primer	Sequence 5′ to 3’
Canine forward	AGGGCGCGATCCTGGAGAC
Canine reverse	AGACACAGGCAGAGGGAGAA

### Statistical analysis

Statistical analyses were performed using Graph Pad Prism 7 software. Alkaline phosphatase levels in each cell line were recorded and a Kruskal-Wallis test with a Dunn’s multiple comparison test was used to compare tumor cell lines to the TNO cell line. Cell line sensitivity to cisplatin was interpolated by calculating the fold change in viability based on a standard curve generated by exposure to cells of varying concentrations. Student’s T tests were used to determine the difference between cells grown in differentiation media and controls. For whole lung DNA analysis known canine and human DNA samples were used to generate sigmoidal standard curve from which all unknown values were determined. Once the unknowns were determined an unpaired t test was used to determine significant differences between treatment and control groups. Invasiveness, degree of oxidative damage, tumor growth rates and the number of mice with reported metastatic lesions were recorded and graphed. Significance was set at a *p* value of 0.05.

## Supplementary information

**Additional file 1: Figure S1.** Differentiation Media in all cell lines. Five and 10X images of the control and differentiating media cells for the various cell lines. This file includes an image of the various cell lines (5 newly characterized ones) as well as 2 previously immortalized cell lines in differentiation medias (adipose, osteogenic and chondrogenic).

## Data Availability

Summaries of the data are provided within the manuscript. All raw data is held by the corresponding author and may be available upon request.

## Abbreviations

ADM: Adipocyte differentiation media
ALP: Alkaline phosphatase stain
AO/PI: Acrid orange/ propidium iodide
CDM: Chondroblastic differentiation media
H&E: Haemotoxyline and eosin stain
MI: Mitotic index
ODM: Osteogenic differentiation media
OS: Osteosarcoma
OST: Overall survival time
PFI: Progression free interval
TNO: Primary normal osteoblast cell line
