# Supplementary figures and images for: Characterization of five newly derived canine osteosarcoma cell lines
Source: BMC Vet Res. 2019 Oct 22;15:357. doi: 10.1186/s12917-019-2099-y (PMC6805340; doi:10.1186/s12917-019-2099-y)

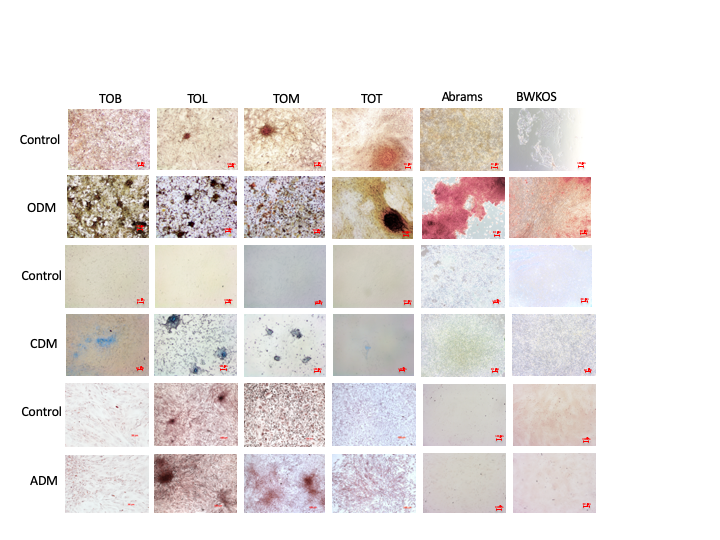

Supplement: Supplementary file 1 — Additional file 1: Figure S1. Differentiation Media in all cell lines. Five and 10X images of the control and differentiating media cells for the various cell lines. This file includes an image of the various cell lines (5 newly characterized ones) as well as 2 previously immortalized cell lines in differentiation medias (adipose, osteogenic and chondrogenic). [file 12917_2019_2099_MOESM1_ESM.tiff]
